# Monthly Record of Deaths in the City of Buffalo

**Published:** 1854-10

**Authors:** J. M. Newman

**Affiliations:** Health Physician


### ART. VII.—*Record of Mortality in the City of Buffalo, for the Month of August*, 1854. By J. M. NEWMAN, Health Physician.

| DISEASES. | No. | Males. | Females. |
|---|---|---|---|
| Accidens, | 6 | 4 | 2 |
| Apoplexia, | 4 | 4 | |
| Asphyxia Immersorum, | 5 | 4 | |
| Cancer Linguæ, | 1 | 1 | |
| " Uteri, | 1 | | 1 |
| Cholera Asphyxia, | 223 | 123 | 95 |
| " Infantum, | 79 | 33 | 39 |
| " Morbus, | 13 | 8 | 5 |
| Cynanche Trachealis, | 1 | 1 | |
| Debilitas, | 16 | 10 | 5 |
| Diarrhœa, | 36 | 19 | 17 |
| Dysenteria, | 30 | 14 | 14 |
| Eclampsia, | 22 | 13 | 9 |
| " Gravidarum et Parturientium, | 1 | | 1 |
| Enteritis, | 2 | | 2 |
| Febris, | 3 | 2 | 1 |
| " Biliosa, | 1 | | 1 |
| " Congestiva, | 1 | 1 | |
| " Intermittens, | 1 | | 1 |
| " Puerperalis, | 1 | | 1 |
| " Scarlatina, | 1 | 1 | |
| " Typhoid, | 8 | 4 | 4 |
| " Typhus, | 9 | 3 | 6 |
| Hydrops, | 3 | 1 | 2 |
| " Abdominis, | 1 | | 1 |
| " Cerebri, | 9 | 6 | 2 |
| Hyperæmia Cerebri, | 7 | 3 | 4 |
| " Pulmonalis, | 1 | | 1 |
| Insania, | 2 | 2 | |
| Intemperantia et Delir. Tremens, | 4 | 3 | 1 |
| Marasmus, | 3 | 1 | 2 |
| Morbi Cordis, | 5 | 2 | 3 |
| Morbus Hepaticus, | 1 | 1 | |
| Natus Mortuus et Partus Abortivi, | 7 | 2 | |
| Negligentia, | 2 | | 2 |
| Perforatio Intestinorum, | 1 | 1 | |
| Peritonitis, | 1 | 1 | |
| Pertussis, | 3 | 1 | 1 |
| Phthisis Pulmonalis, | 20 | 9 | 11 |
| Phrenitis, | 4 | 3 | 1 |
| Pneumonia Typhoid, | 1 | 1 | |
| Rubecla, | 3 | 2 | |
| Scrophula, | 1 | | 1 |
| Senectus, | 5 | 3 | 2 |
| Syphilis, | 2 | | 2 |
| Ignotus, | 36 | 22 | 12 |
| | 587 | | |

| | |
|---|---|
| Males, | 309 |
| Females, | 252 |
| Sex not given, | 26 |
| Total, | 587 |

REGISTER OF MORTALITY—(Continued.)

AGES.

| | | | |
|---|---|---|---|
| Still-born, | 4 | Between 6 years and 12 years, | 31 |
| 1 day, | 4 | " 12 " " 20 " | 33 |
| Between 1 day and 15 days, | 10 | " 20 " " 30 " | 78 |
| " 15 days and 30 days, | 17 | " 30 " " 40 " | 75 |
| " 1 month and 3 months, | 18 | " 40 " " 50 " | 36 |
| " 3 months and 6 months, | 19 | " 50 " " 60 " | 20 |
| " 6 " " 9 " | 28 | " 60 " " 70 " | 16 |
| " 9 " " 12 " | 33 | " 70 " " 80 " | 4 |
| " 1 year and 3 years, | 111 | " 80 " " 90 " | 2 |
| " 3 years and 6 years, | 46 | Ages not given, | 2 |
| Total, | | | 587 |

NATIVITIES.

| | | | |
|---|---|---|---|
| American, | 132 | Italian, | 2 |
| English, | 8 | Swiss, | 6 |
| Scotch, | 4 | Norwegian, | 5 |
| Irish, | 70 | Hollander, | 1 |
| Canadian, | 2 | Colored, | 2 |
| German, | 268 | Country not given, | 80 |
| French, | 7 | | |
| Total, | | | 587 |

In the above tables are included 18 deaths occurring at the Alms House during the month of August, 4 of which were by Cholera.

